# A Rare Case of Neonatal Desmoid Tumor Leading to Severe Aortic Coarctation: Review of Literature and Case Report

**DOI:** 10.3390/life15010123

**Published:** 2025-01-17

**Authors:** Irina Maria Margarint, Tammam Youssef, Cristina Filip, Ana-Mihaela Bizubac, Alexandru Popescu, Iulian Rotaru, Olguta Untaru, Stefan Manolache, Vlad Anton Iliescu, Radu Vladareanu

**Affiliations:** 1 Faculty of Medicine, Carol Davila University of Medicine and Pharmacy, 050474 Bucharest, Romania; irina-maria.margarint@drd.umfcd.ro (I.M.M.); vladanton.iliescu@gmail.com (V.A.I.); vladareanu@gmail.com (R.V.); 2 Department of Cardiac Surgery, Emergency Clinical Hospital for Children “Maria Skłodowska Curie”, 077120 Bucharest, Romania; alexandru.a.g.popescu@gmail.com (A.P.); rotaru.iulian7@yahoo.com (I.R.); untaru_olguta@yahoo.com (O.U.); 3 “Marie S. Curie Children’s Emergency Hospital” Bucharest, Neonatal Intensive Care Unit, 20 Constantin Brancoveanu Street, District 4, 041451 Bucharest, Romania; ana-mihaela.bizubac@umfcd.ro (A.-M.B.); stefan-marius.manolache@drd.umfcd.ro (S.M.)

**Keywords:** desmoid tumor, aortic coarctation, newborn, respiratory failure

## Abstract

Desmoid tumors are a rare entity, especially in the pediatric population. There are no reports of such a tumor in newborns. They are associated with high rates of morbidity and mortality, even though they are benign soft tissue tumors. This is due to them exhibiting locally aggressive growth with the compression or invasion of adjacent structures. Abdominal localization is most commonly reported, although there are reports of mediastinal desmoid tumors. We present the case of a 6-day male patient with a mediastinal desmoid tumor that led to severe aortic coarctation with hemodynamic instability. The tumor also compressed the left pulmonary artery and obstructed the left main bronchus. The initial management consisted of successful emergency surgery with partial resection of the tumor mass and coarctation repair. In the postoperative setting, the patient evolved with severe respiratory dysfunction which was managed with tracheostomy, allowing weaning the child from the mechanical ventilation one month after surgery, along with chemotherapy. We also review the literature, focusing on the management of desmoid tumors.

## 1. Introduction

Desmoid tumors (DTs) are a rare entity with an incidence of 2–4 new cases/1 million/year in the general population [1]. In the pediatric population, the incidence is scarce, and the reported peak incidence occurs between 5 and 8 years [2]. Although they are benign soft tissue tumors, they are associated with high rates of morbidity and mortality because of their locally aggressive growth, resulting in the compression or invasion of adjacent structures [2]. Histopathologically, they are characterized by a proliferation of spindle-shaped fibroblasts arranged in fascicles within a collagen-rich extracellular matrix. These tumors lack a capsule and infiltrate surrounding tissues, often blending with adjacent normal structures. Immunohistochemistry typically reveals the expression of β-catenin, which is a hallmark of these tumors and may also show positivity for vimentin and smooth muscle actin, reflecting their fibroblastic origin. Their mitotic activity is usually low, and there is an absence of significant nuclear atypia or necrosis, distinguishing them from malignant sarcomas [1,2].

Mediastinal DTs located at the level of the mediastinum is a rare finding, especially in the pediatric population [3,4,5]. Anterior chest wall localization is the most frequently reported [3,4] in pediatric patients, and only five cases have been observed with true intrathoracic DTs in this population [5]. There are no reports of a mediastinal DT resulting in aortic coarctation and left main bronchus stenosis in children and there have been no DTs described in newborns.

The active treatments for DTs are surgery, radiotherapy, and systemic treatment. Most of the data regarding treatment strategies in children come from the adult population. There are no guidelines for the management of these tumors, especially those with mediastinal localization. The use of surgery as the initial approach is reported not to be superior to a conservative approach, especially when considering that surgery could result in a significant loss of tissue and function [6].

We reviewed the literature on DTs in the pediatric population, focusing especially on the mediastinal localization and treatment possibilities, and presented a case of a male newborn transferred to our institution 6 days after birth with mediastinal localization of a DT. The DT involved both the aorta and the left main bronchus, causing severe aortic coarctation, significant obstruction of the left main bronchus, and left pulmonary branch stenosis, and was managed with emergency surgery and chemotherapy.

## 2. Case Presentation

**Figure 1:**
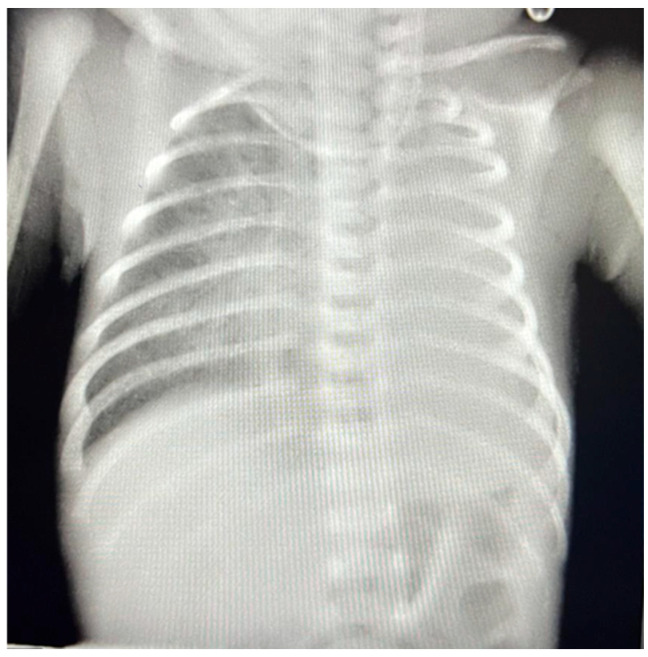
Chest XR showing opacification of the entire left pulmonary field with a retractile effect on adjacent structures.

We present the case of a 6-day-old male transferred to our institute from a tertiary center with the diagnosis of severe aortic coarctation and respiratory failure. The patient was born via cesarean section at a gestational age of 38 weeks with an Apgar score of 8 at birth. In the family history, it is noteworthy that the patient’s mother had thrombophilia. At birth, the patient’s SpO2 remained below 90%, prompting the initiation of free-flow oxygen therapy. At 1 h of life, the patient developed signs of respiratory distress syndrome, with SpO2 levels between 88–92%, leading to the initiation of non-invasive ventilation via BCPAP (flow 7 L/min, PEEP 7.5 cmH2O, FiO2 25–45%), which achieved SpO2 levels of 90–94%. Serial chest X-rays revealed heterogeneous opacification of the entire left pulmonary field with a retractile effect on adjacent structures (Figure 1). An initial transthoracic echocardiography (TTE) revealed severe aortic coarctation and a patent ductus arteriosus (PDA) with bidirectional shunting.

At presentation, a physical examination revealed a newborn of 2800 g, spontaneously breathing in room air, who was tachypneic (respiratory rate was 60–65 breaths per minute) with subcostal retractions and sternal indrawing. Bilateral vesicular breath sounds were present, diminished at the left lung base, without crackles. The preductal SpO2 was 92%, and the postductal SpO2 was 85–88%. A cardiovascular system examination revealed rhythmic and regular heart sounds, a heart rate of 180 bpm, and blood pressure at the right upper limb of 105/61 (79) mmHg, at the left upper limb of 65/32 (43) mmHg, and at the lower limbs of 67/48 (55) mmHg. Also, a grade 4 out of 6 systolic murmur was observed, as well as diminished femoral pulses and a persistent pre- and post-ductal systolic blood pressure difference around 40 mmHg. The abdomen was soft and depressible on palpation. Bowel transit and urinary output were present, and the neurological examination was within normal limits.

The transthoracic echocardiography performed at admission revealed left ventricle systolic dysfunction with an ejection fraction of 35%, normal right ventricular kinetics, no valvular abnormalities, and an intact interventricular septum. At the level of the aortic arch, the following dimensions were observed: a proximal transverse arch measuring 6.5 mm, a 3.3 mm hypoplastic distal transverse arch after the emergence of the left common carotid artery with a diameter of 3.3 mm, and a narrowed isthmus of 1 mm diameter with a systolic pressure gradient of 60 mmHg and holodaiastolic runoff. 

**Figure 2:**
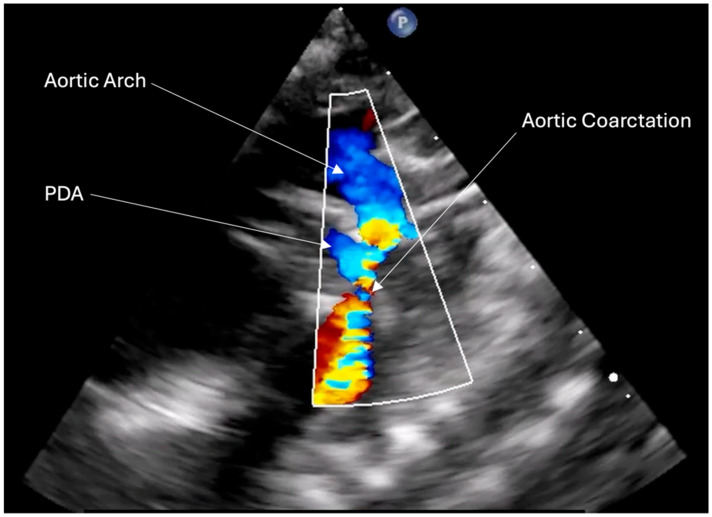
TTE at admission showed severe aortic coarctation and PDA (persistent ductus arteriosus) with right to left shunt.

The left subclavian artery was significantly reduced in size at its proximal end, with turbulent flow. A large patent ductus arteriosus was noted, measuring 4 mm in diameter at the pulmonary end and 2 mm at the aortic extremity, with bidirectional shunting (Figure 2). The abdominal aorta showed reduced pulsatility, with a maximum systolic velocity of 0.3 m/s. Additionally, the left pulmonary artery had normal dimensions at its proximal end, becoming narrowed at its mid-segment (2.8 mm), while the right pulmonary artery had normal dimensions (6 mm) along its entire length. The gradient between the right ventricle and right atrium was 36 mmHg.

**Figure 3:**
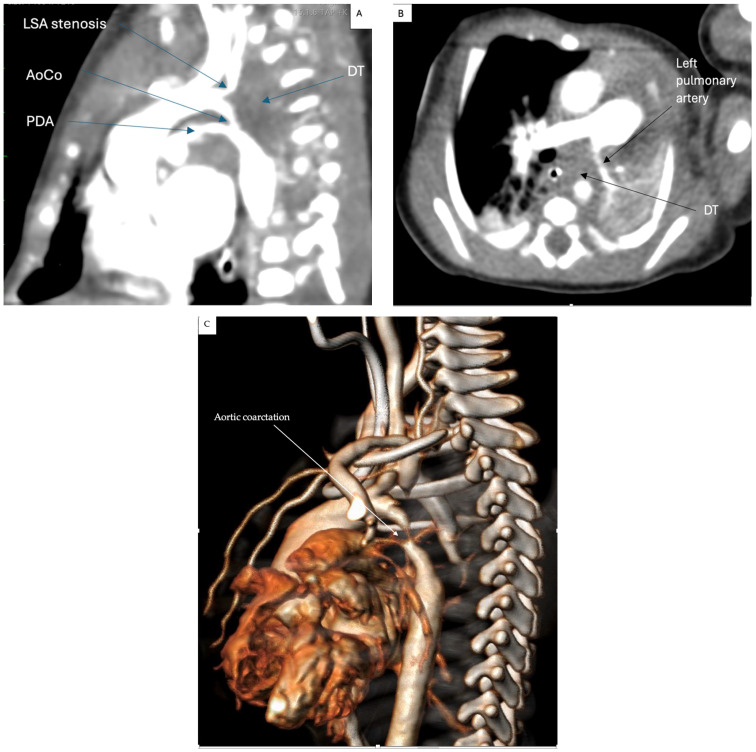
Chest CT angiography. (**A**) LSA: Left subclavian artery, proximal stenosis; CoAo: aortic coarctation; PDA: persistent ductus arteriosus; DT: desmoid tumor; (**B**) desmoid tumor (DT) compressing the left pulmonary artery and invasion of the left main bronchus which cannot be visualized; (**C**) 3D CT reconstruction.

The complete blood count, coagulation tests, and blood biochemistry were within normal limits except for high NTPBNP (69,905 pg/mL) and troponin levels (148 ng/L). The patient’s condition deteriorated rapidly, becoming hypotensive together with worsening respiratory dysfunction (SpO2 below 80% and hypercapnia), oliguria, and acidosis. Inotropic support was initiated with dopamine, PGE1 was continued because of the ductal-dependent circulation, and the initially non-invasive ventilation (MCV) for the management of the respiratory dysfunction was converted to nasotracheal intubation and mechanical ventilation. An emergency thoracic angio-CT was performed, revealing a space-occupying lesion in the superior mediastinum, encasing vascular structures at this level and the left main bronchus. The findings included severe aortic coarctation with a patent ductus arteriosus (PDA), proximal severe stenosis of the left subclavian artery with post-stenotic dilation, and a smaller left pulmonary artery. Additionally, the left lung was hypoplastic, and the tumor mass invaded the left main bronchus, causing severe obstruction (Figure 3). The patient was transferred to the surgical theatre for emergency surgery. We believed that surgical treatment offered more advantages compared to interventional therapy. First, given that the aortic coarctation was caused by tumor invasion/compression, balloon angioplasty would have been a suboptimal method due to the potential for tumor progression over time. Additionally, through tumor excision, we aimed to address both the correction of the aortic coarctation and the severe stenosis of the left main bronchus. Moreover, this strategy provided essential data on the tumor’s histopathology, which is crucial for subsequent oncological management.

### 2.1. Surgical Technique

After the institution of general anesthesia, a left posterolateral thoracotomy with access to the pleural cavity through the third intercostal space was conducted. At the level of the posterior thoracic wall, anterior to the descending aorta, a firm, cartilaginous tumor formation was palpable. This occupied the space between the vertebral column and the pericardial sac, with deviation of the pleuro-pericardial cleft (the phrenic nerve was deviated posteriorly). The tumor formation was gradually resected. The tumor involved the left pulmonary artery, the ductus arteriosus, the isthmic aorta, and, partially, the descending aorta, the left subclavian artery, and the vagus nerve. After careful dissection and resection of the tumor mass, all the above structures were released. Behind the aorta, a portion of the tumor remained intimately adherent to the thoracic wall. The isthmic aorta and the thoracic descending aorta both had a firm consistency, and the left subclavian artery originated from the stenotic segment of firm consistency. Next, we proceeded with double ligation and sectioning of the ductus arteriosus. After systemic heparinization with 200 UI of unfractionated heparin, the aortic arch was clamped proximal to the origin of the left common carotid artery, and the descending aorta was clamped. A longitudinal arteriotomy was made, beginning from the distal aortic arch and extending to the descending aorta. A pericardial patch was sutured to the margins of the arteriotomy in a continuous fashion using polypropylene 7.0. After distal and proximal declamping, we observed egalization of the femoral and brachial arterial pressures. Next, hemostasis was achieved, the lungs were recruited, and a chest tube was inserted in the left pleural cavity. The intercostal space was approximated with absorbable sutures, the chest wall was closed layer by layer, and an intracutaneous suture was performed.

Given the fact that the tumor invaded adjacent structures, including the isthmus and descending aorta, we believed that using a patch aortoplasty technique would be the safer method, as it would not require an extensive mobilization of the descending aorta and aortic arch. Also, the approach used was a left thoracotomy, and enlarging of the left pulmonary artery would have been difficult and more challenging through this approach.

Due to the marked initial hemodynamic instability, a decision was made to perform a patch enlargement angioplasty for the correction of the aortic coarctation, adopting a watchful waiting approach regarding the subclavian artery stenosis. A potential reimplantation of the subclavian artery would have prolonged the operative time and the duration of aortic clamping, thereby increasing the risks associated with the severe preoperative left ventricular dysfunction. Furthermore, the conservative approach that we took with regards to the subclavian artery was also based on the absence of clinical signs of hypoperfusion in the left upper limb. Pericardial patch angioplasty offers several advantages as a treatment for aortic coarctation. This technique provides a durable and anatomically precise repair, minimizing the risk of restenosis compared to other methods. It allows for tailored reconstruction of the aortic arch, particularly in cases with complex anatomies or associated hypoplasia. Furthermore, the use of autologous or bovine pericardium reduces the likelihood of graft rejection and enhances its biocompatibility. This approach also preserves the vascular integrity, avoids the need for prosthetic materials, and facilitates better long-term outcomes, including improved hemodynamic stability and reduced procedural complications.

### 2.2. Postoperative Management

**Figure 4:**
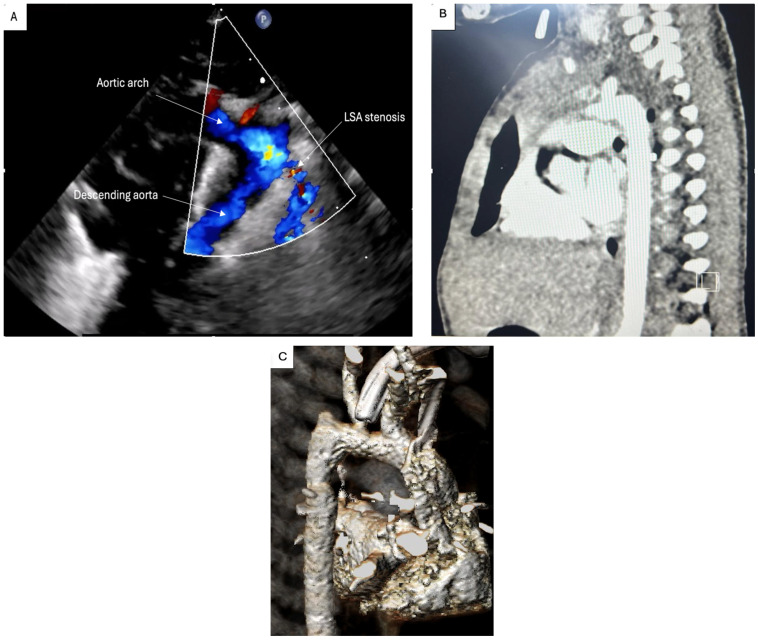
(**A**) Postoperative TTE showing the postoperative result of aortic coarctation repair and proximal left subclavian artery stenosis; (**B**) chest CT angiography showing the result of aortic coarctation repair; (**C**) postoperative 3D CT reconstruction; LSA: left subclavian artery.

In the postoperative setting, the TTE revealed a 6 mm proximal aortic arch and a 5.5 mm distal arch. The aortic isthmus had a diameter of 5 mm, with laminar flow and a maximum systolic velocity of 0.7 m/s. The left subclavian artery had a diameter of 3 mm at its origin and stenosis at 6 mm from the origin with maximum systolic velocity at a level of 2.5 m/s. Moderate left ventricular dysfunction (35% ejection fraction) and also moderate right ventricular dysfunction were observed. The pericardium appeared normal, with no evidence of abnormalities. The abdominal aorta was pulsatile, with a maximum velocity of 0.6 m/s at the diaphragmatic level (and a peak velocity of 8 m/s at the celiac trunk), and persistent arterial flow was observed during diastole (Figure 4A).

Inotropic support with adrenaline 0.03 mcg/kg/min and phosphodiesterase-3 inhibitor (Milrinone, maximum dose of 0.5 mcg/kg/min) was started immediately after the surgery. Nitric oxide was also added in the context of the patient exhibiting postoperative severe respiratory dysfunction with low oxygen saturation. After 3 days, the patient’s hemodynamic status permitted weaning of the pharmacological support. Another chest CT scan was obtained, which showed a residual mediastinal tumor mass encasing the left pulmonary hilum, severe stenosis/collapse of the left main bronchus, severe stenosis of the left pulmonary artery, severe stenosis of the left subclavian artery, and hypoplasia of the left lung (Figure 4B).

The patient developed postoperative hypoxemic respiratory failure due to the presence of an intrapulmonary shunt. The ventilation/perfusion mismatch resulted from both partial obstruction of the left bronchus caused by tumor compression and a reduced gas exchange surface due to left lung hypoplasia. This mismatch was partially corrected postoperatively following tumor excision and partial decompression of the bronchus was achieved through peribronchial tumor resection. Subsequent management included the creation of a tracheostomy two weeks after surgery, alternating sessions of non-invasive ventilation, and spontaneous breathing, leading to a gradual but complete correction of the arterial blood gases. Starting from postoperative day 45, the patient started to breathe spontaneously through the tracheostomy cannula with minimal oxygen requirements. The cannula was removed one week prior to discharge at 62 days post-surgery. Chest CT scans at 6 months and at 1 year revealed no progression of the DT. Also, the left upper limb developed normally and showed no signs of hypoperfusion.

**Figure 5:**
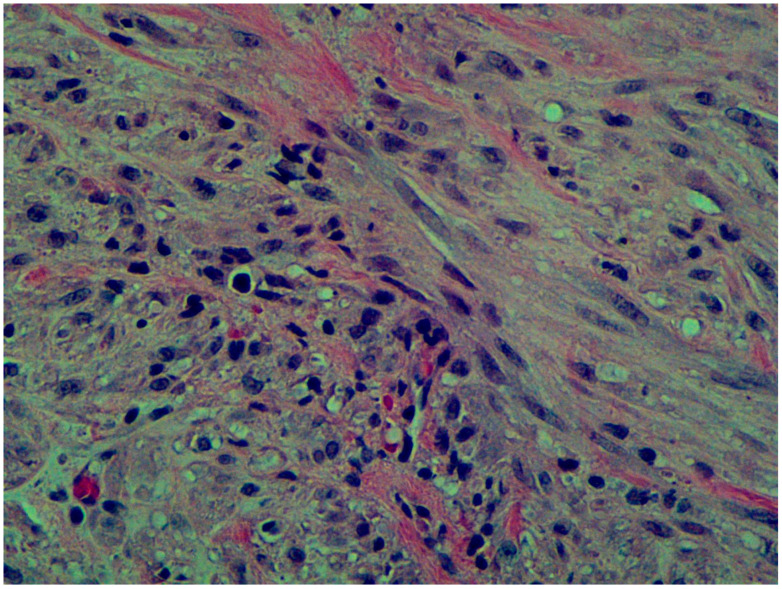
Microscopic examination of the lesion reveals a spindle cell tumor characterized by an interlacing fascicular arrangement of elongated myofibroblastic cells. The tumor cells exhibit elongated, vesicular nuclei with mild pleomorphism and eosinophilic cytoplasm. A prominent perivascular pattern is evident, with tumor cells concentrically arranged around thin-walled capillaries. The stroma is variably collagenous, with focal myxoid areas providing a contrasting background to the more cellular zones. Scattered mitotic figures are observed but remain within a benign range. No significant necrosis or atypical features are noted in this field, and the findings are consistent with infantile myofibromatosis.

The anatomopathological examination raised suspicion of a desmoid tumor, with the final diagnosis being infantile myofibromatosis (Figure 5). Although the mediastinal tumor was benign, considering its compressive effect on adjacent structures, the decision was made, in collaboration with the pediatric oncology team, to initiate “low-dose” chemotherapy with vinblastine (6 mg/m^2^ or 0.2 mg/kg/dose) and methotrexate (30 mg/m^2^ or 1 mg/kg/dose). The patient received weekly cycles of chemotherapy (20 cycles completed) and exhibited good hematological tolerance.

**Figure 6:**
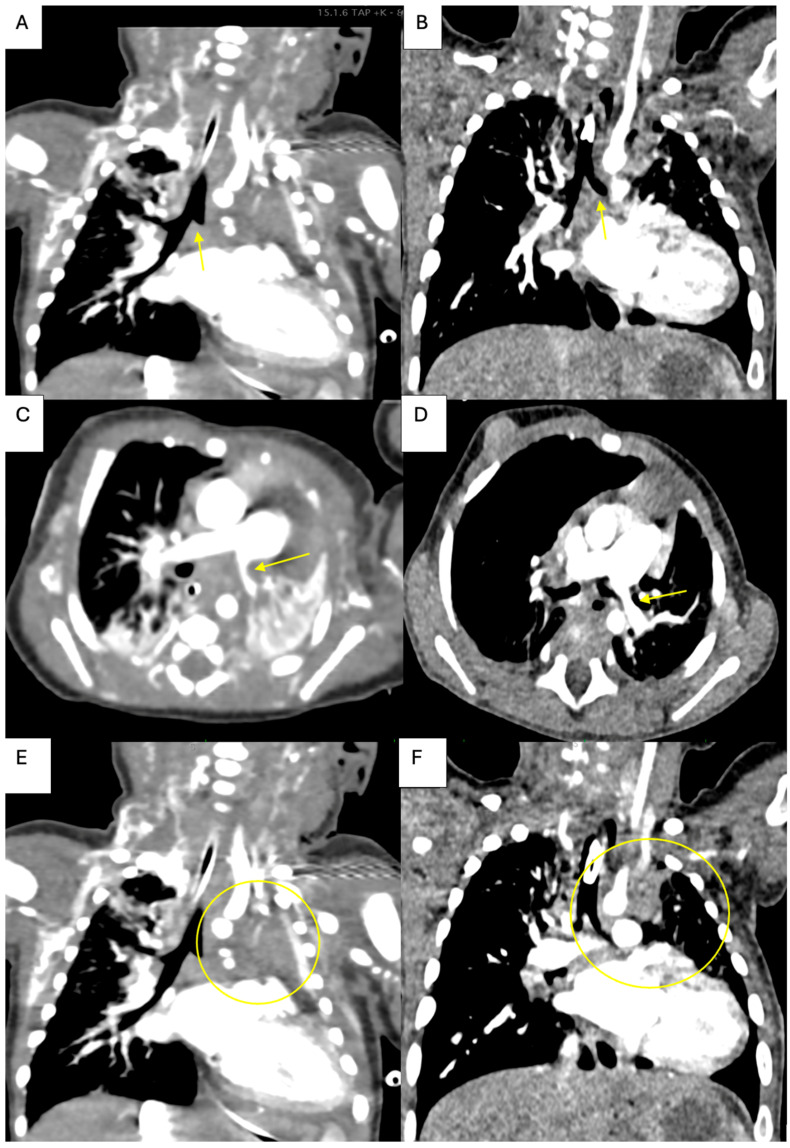
Chest CT comparison at 12 months vs. preoperative; (**A**, arrow) preoperative severe proximal left bronchus stenosis; (**B**, arrow) moderate left main bronchus proximal stenosis at 12 months; (**C**, arrow) preoperative severe proximal left pulmonary artery stenosis; (**D**, arrow) moderate proximal left pulmonary artery stenosis at 12 months; (**E**, circle) preoperative superior mediastinal tumor mass; (**F**, circle) no tumor mass can be seen at the level of superior mediastinum at 12 months.

A follow-up at 12 months included a chest CT scan and TTE. The clinical examination was within normal limits, and the left upper limb was normal despite having no pulse. The TTE showed a normal aortic arch without stenosis with an 11 mm diameter at the level of the patch. The abdominal aorta was pulsatile with a peak velocity of 1 m/s. The severe stenosis at the origin of the subclavian artery was still present. The heart had a normal biventricular function, and the proximal severe stenosis on the left pulmonary artery was now moderate with a 4.5 mm diameter. Compared with the postoperative CT scan (Figure 6), there was no residual mediastinal tumor mass encasing the left pulmonary hilum, as was the case immediately after surgery. Also, the left main bronchus was visible with a mild stenosis. The moderate left pulmonary artery stenosis was also confirmed by TEE. Confirming the TTE result, the left pulmonary artery had moderate stenosis.

## 3. Review

Desmoid tumors (DTs) are a rare entity, with an incidence of 2–4 cases per million of the population per annum, and with a peak age of 30–40 years [7,8]. DTs are benign soft tissue tumors associated with locally aggressive monoclonal, fibroblastic proliferation and characterized clinically by a variable and often unpredictable course [9,10]. The “malign nature” of this type of tumor results from their locally aggressive nature that leads to compression and invasion of adjacent structures.

In the pediatric population, the incidence is scarce, and the reported peak incidence is between 5 and 8 years [2]. A Finnish study of 89 patients identified four distinct peak periods: the juvenile DT described as an extra-abdominal tumor found in young girls, the fertile variety, encountered as an abdominal tumor in women, the middle-age variety described as an abdominal tumor with a similar incidence between males and females and most prevalent in the old age group both, and abdominal and extra-abdominal tumors with a similar sex ratio [1].

DTs can be intraabdominal, within the abdominal wall, or extraabdominal. Up to 69% of DTs are intraabdominal (mesenteric or pelvic) or are located at the level of the abdominal wall. Extraabdominal desmoids are localized in muscle and fascia, and are commonly located at the level of the shoulder and upper extremity (33%), while 30% are located at the level of the gluteal region and lower extremity and 10% at the head and neck region.

A mediastinal DT located at the level of the mediastinum is a rare finding, especially in the pediatric population. Endara et al. [3] reported a case of a DT located in the anterior chest wall in a 6-month-old male. Nadbrzeżna et al. [4] described a case of a DT also located in the anterior chest wall with intrathoracic invasion in a 12-year-old boy. Mayerson et al. [5] reported 22 cases of true intrathoracic DTs, with 12 cases being primarily intrapleural and 10 cases originating in the mediastinum. Only five cases were in the pediatric population. Sporadic cases are described in the adult population (Table 1), with most cases being located at the chest wall with various intrathoracic extensions. We could not find any report of a mediastinal DT resulting in aortic coarctation and left main bronchus stenosis. Also, there are no reports describing DTs in the newborn population.

The etiology of DTs is unclear. Previous trauma is associated with 25% of the cases, with another potential cause being familial adenomatous polyposis (FAP), especially Gardner Syndrome. In the absence of familial adenomatous polyposis, familial groups of DTs also exist and are linked with the adenomatous polyposis coli gene on chromosome 5. β-catenin gene mutations and adenomatous polyposis coli gene alterations have been linked to sporadic and FAP-associated DT. A hormonal influence is also linked to DT occurrence. High levels of estrogen and antiestrogen binding sites are often identified in cases of DTs and many series note an increased incidence in women of reproductive age [5,11].

The active treatments for DT are surgery, radiotherapy, and systemic treatment. Even though most of the data regarding treatment strategies come from the adult population, many of these findings have been applied to pediatric patients. Also, it should be mentioned that all the studies comparing different therapy strategies are classified as “very low” according to GRADE.

### 3.1. Surgery Compared to Observation

When considering surgery as the initial approach, there was no difference regarding event-free survival at 2 years (EFS) and long-term disease control when compared to a conservative approach in 771 confirmed cases of DT (53% versus 58%; *p* = 0.415), according to Penel et al. [6]. The location of the tumor seems to play a role in the disease course [12,13,14]. Favorable locations like abdominal wall, intra-abdominal, breast, lower limb, and digestive viscera have a similar 2-year EFS between surgery and conservative groups. The 2-year EFS was better in the non-surgical group for DTs localized at the level of the chest wall, head and neck, and upper limbs. The non-superiority of the surgical strategy regarding EFS is also sustained by Salas et al. [15] in a study of 426 patients with a DT, which included 46 pediatric patients. In this study, no intrathoracic DT was described. Abdominal wall or extra-abdominal localization seems to benefit more from a conservative strategy with a low incidence of conversion for this strategy at 5 years [16,17]. In pediatric patients, the conservative strategy was superior when compared to the surgical strategy in terms of EFS according to Park et al., who conducted a study of 47 pediatric patients [18]. Also, in patients with FAP, the survival at 10 years was comparable between the non-surgical and surgical strategies [19]. In conclusion, the 2019 guidelines from the European Reference Network for Rare Solid Adult Cancers and The Desmoid Tumour Working Group recommend the initial observation of asymptomatic patients, independently of the tumor site, in the setting of an experienced team in a connective tissue tumors center [20].

The recurrence of DTs plays a significant role in the long-term prognosis of pediatric patients. Soto reported 1- and 5-year recurrence-free survival rates of 97.1% and 73.1% in 39 patients with a mean age of 12.2 years who were treated with surgery [21]. Risk factors for recurrence were an age over 12 years and a tumor size of more than 5 cm, irrespectively of the status of the resection margin. Mullen reported a 60% 10-year recurrence rate in 177 adult patients with surgery for DTs and stated that half of the patients with positive margins will experience recurrence; thus, negative margins should be the goal [22]. Positive margins were predictive of the recurrence of the primary tumor, especially in extra-abdominal desmoids, in a study of 198 adult patients reported by Huang et al. [23]. In pediatric patients, negative margins should be the goal, as this is reported to be the only factor associated with recurrence-free patients in a study of 63 cases with a mean age of 13 years [24].

The role of adjuvant-like radiotherapy remains unclear. Different retrospective studies failed to show a significant difference between surgery alone and surgery plus radiotherapy in terms of recurrence risk reduction. Adding radiotherapy can be an approach to recurrent disease if surgery is an option but the risk of radiation-induced sarcoma should be taken into consideration in the pediatric population [25,26,27,28].

### 3.2. Nonsurgical Strategies

Radiotherapy (RT) is another strategy for the treatment of DTs, especially in symptomatic tumors located in critical sites such as the head and neck. Radiotherapy alone has a similar risk of recurrence compared to radiotherapy associated with surgery [29,30]. Guadagnolo et al. reported that DTs were effectively controlled with RT administered either alone or as an adjuvant to surgery when the resection margins were positive in a study of 115 patients, which included pediatric patients [29]. The study reported a 74% rate of no recurrence at 10 years. Spear et al. confirmed the findings of Guadagnolo et al. in 117 patients with DT, which also included pediatric patients [30]. They reported a 100% disease-free rate at 5 years when surgery and RT were the first strategies used and stated that RT is an effective alternative in situations where surgery would result in major functional defects. Analyzing the risk of progressive disease, there was no significant difference between the use of initial RT and the use of initial surgery [31]. However, the author states that gross total resection should be the first strategy if it can be performed without significant disfigurement, and that RT should be indicated in patients with recurrent disease to obtain local control.

Systemic treatment options are anti-hormonal therapy (AHT), non-steroidal anti-inflammatory drugs (NSAIDs), tyrosine kinase inhibitors (TKIs), and chemotherapy.

Some studies report an up to 25% response rate of AHT coupled with NSAIDS therapy [32,33,34]. However, the only phase II study in the pediatric population reported limited activity according to World Health Organization response criteria. Skapek et al. enrolled 59 pediatric patients with DT, 22 cases of naïve patients, and 37 patients with recurrent disease. In the recurrent disease group, 6 had previous chemotherapy, and 15 had prior radiotherapy. The treatment consisted of tamoxifen and sulindac. Only 10 patients completed the one-year regimen without disease progression, partial regression was observed in 4 patients, and complete response to therapy was encountered in 1 patient. The overall survival rate was 96% [35].

Different TKIs were evaluated for the treatment of DT: imatinib, nilotinib, sorafenib, and pazopanib. There are no studies in the pediatric population regarding the use of TKIs. Chung et al. [36] reported a 66% progression-free survival (PFS) rate and an overall response rate (ORR) of 6% in 51 adult patients with unresectable DT treated with imatinib. Penel et al. [37] report better results (PFS of 91%, 80%, and 67% at 3, 6 and 12 months and an ORR of 11%) with imatinib in 35 adults with unresectable DT, while the German Interdisciplinary Sarcoma Group [38] communicates a 65% progression arrest rate and an ORR of 19% in 38 adult patients with tumor progression. In conclusion, imatinib had a high rate of DT stabilization but a low response rate. Gounder et al. [39] studied sorafenib and reported an ORR of 33% in 87 adult patients, but used a weak study design that included patients with a modest increase in tumor size and no guidelines to assess symptoms of severity, including patients that probably did not necessitate active treatment. Pazopanib had a 6-month non-progression rate of 82% with a response rate similar to that of sorafenib, suggesting that these two can be of benefit in the treatment of DTs [40].

There are two chemotherapy options: a “low-dose” regimen with methotrexate and vinblastine or a conventional dose of anthracycline-based regimens. Studies evaluating the first option in adults report a response rate from 35% to 40% several months after the start of the regimen, with long-term disease control achieved in from 50% to 70% [41,42]. A more rapid response is achieved with conventional dose chemotherapy using anthracycline-based regimens, with a 37% response rate. De Camargo et al. [43] and Garbay et al. [44] reported similar response rates in the pediatric population.

In conclusion, The Desmoid Tumor Working Group recommends taking into consideration, besides the level of evidence, overall response, and PFS rate, the expected toxicity, because of the lack of comparative studies. Prospective phase II studies exist only for low-dose chemotherapy with methotrexate and vinblastine and for the use of imatinib. Their recommendations are to administer aggressive therapies only in worst-case scenarios with a life-threatening DT [45].

An unresolved issue in the treatment of DTs is monitoring the treatment effect. Contrast-enhanced MRI, CT, and fluorodeoxyglucose FDG-PET are currently the preferred modalities. Circulating tumor DNA could have a role in the management of DTs and is currently under evaluation as a biomarker of response/progression [45,46,47,48].

### 3.3. Particularities of Treatment of Aortic Coarctations in Newborn

Aortic coarctation in neonates requires a specialized approach to treatment due to unique anatomical and physiological factors specific to this age group. Neonates often present with critical coarctation, leading to severe left ventricular afterload and heart failure that prompts immediate management. Particularities found in neonates include the following: ductal dependency, fragility of tissues, associated anomalies, and rapid recovery needs [49]. Many neonates rely on a patent ductus arteriosus for systemic blood flow. This requires careful timing of the intervention, often necessitating preoperative prostaglandin therapy to stabilize the neonate, while meticulous fluid and temperature management are needed to ensure optimal recovery, considering the neonates limited physiological reserves. Also, the small size and fragility of neonatal vessels increase the risk of complications during both surgical and interventional procedures, especially since neonates frequently have coexisting congenital heart defects, such as ventricular septal defects or hypoplastic aortic arches, which must be addressed simultaneously during treatment [50].

Extended end-to-end anastomosis remains the preferred treatment for neonatal coarctation, as it addresses both the narrowed segment and associated aortic arch hypoplasia [51]. In neonates, the elasticity of the aortic tissue facilitates tension-free anastomosis, which is crucial for preventing restenosis. The surgical approach often involves median sternotomy or lateral thoracotomy, depending on the associated cardiac anomalies. Due to their immature physiological status, neonates require meticulous perioperative management to maintain hemodynamic stability and adequate oxygenation.

Although less invasive, balloon angioplasty is less commonly employed as a primary treatment in neonates due to a higher risk of complications, such as vascular rupture and aneurysm formation [18,52,53,54,55,56]. It is typically reserved for patients with contraindications to surgery or used as a bridge therapy in hemodynamically unstable patients. In neonates with ductal-dependent systemic circulation, angioplasty may be combined with prostaglandin infusion to maintain ductal patency until definitive surgical repair is possible.

## 4. Conclusions

The management of mediastinal desmoid tumors in newborns is challenging because of the rare incidence in this population, as well as the high mortality and morbidity, and the frail condition of these patients. The evolution of this type of tumor with local invasion of the superior mediastinum structures in a 6-day-old newborn, which lead to severe aortic coarctation, obstruction of the left main bronchus, and severe stenosis of the left pulmonary artery and left subclavian artery, presented a unique association with a probability of high mortality. There are no guidelines for the management of desmoid tumors. Although different studies report that surgery is not superior to a conservative strategy or chemotherapy/radiotherapy, in our case, prompt emergency surgery was needed because of the severe aortic coarctation, the ineffectiveness of the patent ductus in ensuring systemic flow, and the hemodynamic instability after birth. Invasion of the left main bronchus and pulmonary artery lead to respiratory failure in the postoperative setting, demonstrating the high morbidity of the mediastinal localization of this tumor. The particularity of this case is the development of the tumor during fetal life, this condition being responsible for the incomplete development of affected vascular and respiratory structures. Therefore, we can talk about two mechanisms involved in respiratory and vascular damage: a lack of proper development of these structures during fetal life (hypoplasia of left lung, pulmonary branch, and left bronchia) and the direct compressive effect of tumoral mass over these structures. Besides this, compared with usual aortic coarctation, in our case, maintaining patent ductus arteriosus by prostaglandin infusion was useless because the juxtaductal aortic segments and the aortic extremity of the ductus were caught in the tumoral mass, thus not allowing perfusion of the descending aorta through the ductus.

A patient-tailored strategy should always be adopted in these cases, especially in the mediastinal localization, considering that surgery for removing the desmoid tumor could result in a significant loss of tissue and function because of the locally aggressive growth.

## Figures and Tables

**Table 1:** Mediastinum localization reports of DT.

	Age/Sex	Year	Primary Location	Extension	Management
Gupta et al. [11]	36 years/female	2005	Posterior mediastinum	Left lower pulmonary lobe	Resection
Meyerson et al. [12]	5–66 years	2008	12 cases Intrapleural and 10 cases in the medisatinum	NS	Resection
Kocak et al. [13]	40 years/male	2000	Posterior mediastinum	Transdiaphragmatic extension	Inoperable
Takeshima et al. [14]	46 years/female	2001	Left chest wall	Pleural cavity	Resection
Murakava et al. [15]	39years/women	2008	Chest wall	NS	Resection
Hashizume et al. [16]	75 years/women	2007	Chest wall	NS	Resection
Sakamoto et al. [17]	18 years/female	2001	Apex of the chest wall	Brachial plexus	Resection
Kaplan et al. [18]	18 years/male	1986	Right lung	Esophagus and diaphragm	Resection

NS: not specified.

## Data Availability

Data are available upon request.
